# Extract from the Report of the Superintendent of the Buffalo General Hospital

**Published:** 1867-01

**Authors:** Wm. C. Bagley

**Affiliations:** Superintendent; Buffalo General Hospital


### Extract from the Report of the Superintendent of the Buffalo General Hospital.

BUFFALO GENERAL HOSPITAL,
December 31st, 1866.

*To the Board of Trustees:*

GENTLEMEN:—I have the honor to submit the following report:

| | |
|---|---|
| Total number of Patients treated since the opening of the Hospital, July 15, 1858 | 2,894 |
| Number as per last annual report | 2,498 |
| Admitted during the year | 396 |
| Males, 329; Females, 67. | |
| In Hospital January 1st, 1866 | 60 |

| | | |
|---|---|---|
| Number of patients under treatment during the year: | | |
| County Patients | 145 | |
| Paying do | 96 | |
| Discharged Soldiers | 101 | |
| Sailors | 49 | |
| Emigrants | 18 | |
| Free | 47 | |
| Number of Medical cases, 321; number of Surgical cases, 135. | —— | 456 |
| Number of Patients discharged well | 275 | |
| " " " relieved | 89 | |
| " " " not relieved | 27 | |
| " " " dead | 33 | |
| | —— | 424 |
| Number of Patients remaining December 31st, 1866 | | 32 |
| Males, 24; Females, 8. | | |
| Proportion of deaths to number of Patients treated, 7.26 per cent. | | |
| Average number of day's Patients remaining in Hospital | | 35 |

The Hospital has been conducted on tbe same plan as the previous year, and it has been the endeavor of its managers to keep the standard high in all respects. Although the number treated was somewhat smaller than during the last year, the results have been equally satisfactory.

High prices were maintained until near the close of the year, and are still in some departments. There is every prospect, however, of a speedy decline in prices, and no doubt the expense of maintaining patients the present year will be materially less than last.

In purchasing for the Hospital I have labored under the disadvantage of lack of ready money—some bills running many months before they are paid. I trust the present year will see the Hospital so far relieved from embarrassment as to enable your buyer to take advantage of low prices in making purchases. By so doing the cost of maintaining the institution would be reduced, and we be enabled to keep private patients at a lower rate, which would have the effect of adding largely to their number.

The expenditures were less in nearly every department than during the previous year, the large exception being the item of buildings and grounds caused by the new sewer, and the paving of High street. But for this, the expenditures would not have been so heavy as the previous year's by nearly eight thousand dollars.

The buildings are in good repair, the Hospital well furnished, and I hope to see our number of patients increased during the year.

The thanks of the Board are due to the Water Company for the free use of their hydrants, and could a valuation be put upon the privilege the amount would be placed in the list of donations.

Early in the year the Buffalo Branch of the Christian Commission placed in the hands of the Trustees twenty-five hundred dollars to be used for the care of discharged soldiers. Of this fund about five hundred dollars remain.

In presenting this report of the third year of my engagement, I would return my sincere thanks to the retiring Board of Trustees for the courtesy and kindness which, personally and officially, I have always received at their hands. To the members of the Medical and Surgical staff, and their representatives, Drs. Shurley and Petit, the resident physicians of the past year, very much is due for the present efficiency of the Hospital.

Respectfully submitted,

WM. C. BAGLEY, SUPERINTENDENT.

